# T-Cell/Histiocyte-Rich Large B-Cell Lymphoma of the Thyroid

**DOI:** 10.1186/2162-3619-2-1

**Published:** 2013-01-09

**Authors:** Satoshi Ichikawa, Yasuaki Watanabe, Kei Saito, Jun Kimura, Ryo Ichinohasama, Hideo Harigae

**Affiliations:** 1 Department of Hematology, Yamagata City Hospital Saiseikan, 1-3-26 Nanukamachi, Yamagata, 990-8533, Japan; 2 Department of Hematology and Rheumatology, Tohoku University Graduate School of Medicine, Sendai, Japan; 3 Clinical Training Center, Yamagata City Hospital Saiseikan, Yamagata, Japan; 4 Department of Hematopathology, Tohoku University Graduate School of Medicine, Sendai, Japan

**Keywords:** T-cell/histiocyte-rich large B-cell lymphoma, Thyroid lymphoma, R-CHOP

## Abstract

T-cell/histiocyte-rich large B-cell lymphoma (THRLBCL) is a rare subtype of diffuse large B-cell lymphoma, and has been reported to mainly affect lymph nodes with advanced Ann Arbor stage. We present a 69-year-old woman with a past history of chronic thyroiditis, who had suffered from rapidly-growing thyroid tumor. No other lymph nodes swelling or extranodal lesions were detected. She promptly underwent surgery with resection of the thyroid tumor, and the diagnosis of THRLBCL was established pathologically. She was successfully treated by standard rituximab-containing chemotherapy. To our knowledge, this is the first case report of THRLBCL exclusively arising in the thyroid.

## To the editor

**Figure 1:**
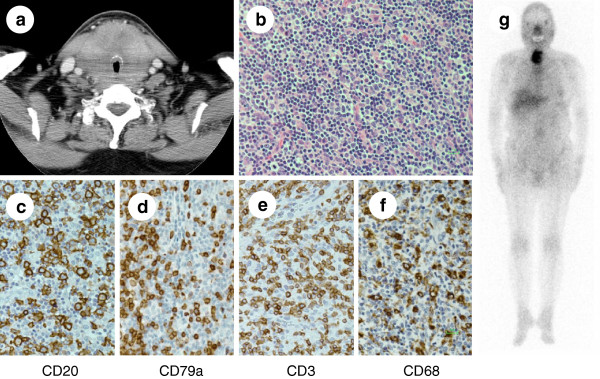
**(1a) Computed tomography of the thyroid tumor. (1b)** Microscopic examination of the thyroid revealed that the tumor is comprised of scattered large atypical cells embedded in a background of histiocytes and small lymphocytes (hematoxylin and eosin staining). **(1c,1d)** The scattered atypical cells were positive for CD20 **(1c)** and CD79a **(1d)**. **(1e)** Small lymphocytes represent CD3-positive T-cells. **(1f)** Substantial numbers of CD68-positive histiocytes are also intermingled. **(1g)** Gallium scintigraphy showed increased gallium uptake in the thyroid exclusively.

A 69-year-old woman was admitted to our hospital due to difficulty in breathing and thyroid tumor that had grown rapidly over a period of 1 month. The patient had a past history of chronic thyroiditis, which was diagnosed a year before on the basis of positive anti-thyroglobulin antibody and elevation of thyroid stimulating hormone with enlarged thyroid. The disease had been well controlled with levothyroxine supplementation. Computed tomography (CT) scan showed a diffuse and massive thyroid tumor narrowing the tracheal lumen (Figure 1a). No other tumors, lymphadenopathy, or hepatosplenomegaly were detected. There were no B symptoms. Due to the risk of sudden airway obstruction, urgent hemithyroidectomy and tracheal tube insertion were performed. Rapid frozen section diagnosis of the resected thyroid tumor suggested the possibility of lymphoma, and administration of prednisolone (PSL) was performed after the operation. The remaining thyroid tumor had reduced to some extent, and the patient’s general condition and respiratory state recovered well. Thorough pathological examination of the thyroid tumor revealed scattered neoplastic large B-lymphocytes, which were CD20^+^, CD79a^+^, CD5^−^, CD10^−^, CD15^−^, and CD30^−^, and surrounding abundant T-lymphocytes (CD3^+^) and histiocytes (CD68^+^) (Figure 1b – 1f). In situ hybridization for Epstein-Barr virus-encoded RNA (EBER) was negative. According to the above findings, the diagnosis of T-cell/histiocyte-rich large B-cell lymphoma (THRLBCL) was established. Gallium scintigraphy showed increased gallium uptake in the thyroid with no other abnormal signals (Figure 1g). Bone marrow aspiration/biopsy revealed no infiltration of lymphoma cells. Serum lactate dehydrogenase was within the normal limits (217 IU/L), and soluble interleukin-2 receptor was slightly elevated (639 U/mL). The clinical stage was IE, and the patient was classified as being at low-intermediate risk according to the international prognostic index. She was successfully treated by chemotherapy consisting of rituximab, cyclophosphamide, doxorubicin hydrochloride, vincristine, and PSL (R-CHOP) without any major issues including tracheal problems. Complete response was confirmed after the completion of six courses of R-CHOP regimen.

THRLBCL is a rare morphological subtype of diffuse large B-cell lymphoma (DLBCL), which is characterized by a limited number of scattered, large, atypical B-cells embedded in a background of abundant T-cells and frequently histiocytes [1]. THRLBCL has been reported to mainly affect lymph nodes, and most cases are at advanced Ann Arbor stage with intermediate to high risk of IPI [1-3]. Although extranodal involvement of THRLBCL is not uncommon, an exclusively extranodal presentation at diagnosis is extremely rare; Weshi et al [2] reported that all of 61 cases investigated had lymph node lesions. In contrast to these observations, our case had a single thyroid tumor with an IPI score indicating low-intermediate risk. To our knowledge, this is the first case report of THRLBCL exclusively arising in the thyroid.

Malignant lymphoma is a minor component of thyroid tumors, accounting for only 2 to 5% of all thyroid malignancies. Thyroid lymphoma is often associated with chronic thyroiditis, and mostly diagnosed as DLBCL and MALT lymphoma pathologically [4]. Also in this case, the past history of chronic thyroiditis might be associated with the emergence of lymphoma.

THRLBCL is considered an aggressive lymphoma. It has also been reported that THRLBCL with histiocytes could represent a very aggressive lymphoma [1]. Although the biological characteristics seem to be different from DLBCL and the effects of rituximab for THRLBCL remain to be determined, it is recommended at present that THRLBCL should be treated similar to stage-matched DLBCL [2]. On the other hand, localized extranodal DLBCL is often treated with radiation therapy with or without chemotherapy, as reported in gastric lymphoma [5]. In this case, however, a tracheotomy was done before lymphoma treatment and radiation therapy could cause airway trouble; so that the patient was treated with R-CHOP chemotherapy without radiation. The patient described here showed a good clinical course; however, careful observation is indispensable to monitor disease relapse at both the primary site and distant sites.

## Competing interests

All authors report having no conflicts of interest to declare.

## Authors’ contributions

SI, First Author and Corresponding Author, was in charge of the patient’s treatment, collected the patient information, reviewed the literature, and drafted the manuscript, and revised the final manuscript. YW and KS assisted in treating the patient and collecting the relevant clinical data. JK was responsible for the patient’s treatment. RI stained and interpreted the pathological slides. HH, Last author, contributed with discussion and review of the manuscript. All authors read and approved the final manuscript.
